# Effect of different aged cartilage ECM on chondrogenesis of BMSCs *in vitro* and *in vivo*

**DOI:** 10.1093/rb/rbaa028

**Published:** 2020-08-04

**Authors:** Xiuyu Wang, Yan Lu, Wan Wang, Qiguang Wang, Jie Liang, Yujiang Fan, Xingdong Zhang

**Affiliations:** 1 Guangxi Engineering Center in Biomedical Materials for Tissue and Organ Regeneration, Guangxi Collaborative Innovation Center for Biomedicine, Guangxi Medical University, Nanning 530021, China; 2 National Engineering Research Center for Biomaterials, Sichuan University, Wangjiang Road 29, Chengdu 610064, China

**Keywords:** cartilage ECM, mesenchymal stem cells, collagen hydrogel, chondrogenesis, calcification

## Abstract

Extracellular matrix (ECM)-based biomaterials are promising candidates in cartilage tissue engineering by simulating the native microenvironment to regulate the chondrogenic differentiation of bone marrow mesenchymal stem cells (BMSCs) without exogenous growth factors. The biological properties of ECM scaffolds are primarily depended on the original source, which would directly influence the chondrogenic effects of the ECM materials. Despite the expanding investigations on ECM scaffolds in recent years, the selection of optimized ECM materials in cartilage regeneration was less reported. In this study, we harvested and compared the articular cartilage ECM from newborn, juvenile and adult rabbits. The results demonstrated the significant differences in the mechanical strength, sulphated glycosaminoglycan and collagen contents of the different aged ECM, before and after decellularization. Consequently, different compositional and mechanical properties were shown in the three ECM-based collagen hydrogels, which exerted age-dependent chondrogenic inducibility. In general, both *in vitro* and *in vivo* results suggested that the newborn ECM promoted the most chondrogenesis of BMSCs but led to severe matrix calcification. In contrast, BMSCs synthesized the lowest amount of cartilaginous matrix with minimal calcification with adult ECM. The juvenile ECM achieved the best overall results in promoting chondrogenesis of BMSCs and preventing matrix calcification. Together, this study provides important information to our current knowledge in the design of future ECM-based biomaterials towards a successful repair of articular cartilage.

## Introduction

Due to the avascular nature, articular cartilage has very limited self-repair capability after damage or degeneration. For many years, surgeons have employed many clinical strategies in attempted to repair cartilage but normally resulted in the regeneration of mechanically inferior fibrocartilage tissues. In recent years, tissue engineering-based techniques using autologous chondrocytes have been introduced and achieved satisfactory results in the clinic. However, due to the limited donor supply and low expansion capacity of chondrocytes, mesenchymal stem cells (MSCs) with the better accessibility and ability to differentiate into chondrocyte-like cells has become the popular alternative. Therefore, to achieve successful cartilage regeneration, it is the key to develop a tissue engineering scaffold that can effectively promote the chondrogenic differentiation of MSCs into functional chondrocytes.

To date, many types of biomaterials, with or without specific biological factors, have been reported to promote the chondrogenesis of MSCs, but the long-term results were always uncertain. To achieve an effective chondrogenic differentiation, MSCs should be placed in a tissue engineering scaffold that mimics the natural environments. Among current selections of biomaterials, synthetic biomaterials have attracted much attention because of their mechanical controllability and wide application prospects [1], but they normally couldn’t reproduce the complicated natural extracellular matrix (ECM) of cartilage, and consequently resulted in the production of inflammatory reaction due to poor degradability [2]. In recent years, ECM scaffolds derived from decellularized natural tissues have been rapidly expanding. With eliminating immunogenicity by removing DNA and preserving the majority of natural ECM components, ECM scaffolds could better simulate the natural environments to regulate MSCs’ differentiation than synthetic scaffolds and have been widely used in clinical treatment, including the trachea [3], heart valve [4], muscle [5], dermis [6], ligament [7] and cartilage [8].

In cartilage tissue engineering, ECM scaffolds have been reported to facilitate the chondrogenic differentiation of bone marrow MSCs (BMSCs) without exogenous growth factors [9]. The decellularized scaffold preserves the structure of cartilage ECM, which provides both mechanical and chemical similarities to natural environments to facilitate the recruitment and differentiation of stem cells while avoiding undesirable biological and immune responses from cellular and nuclear materials [10]. At present, most of the current studies are focussing on the fabrication techniques to convert ECM to highly functional biomaterial scaffolds in cartilage tissue engineering [11]. The decellularization techniques were continuously optimized to preserve more ECM components [12]. The advanced architecture designs of ECM scaffolds were also introduced to promote cell infiltration [13]. Moreover, various mechanically strong biomaterials [14] and chondrogenic growth factors [15] were incorporated in ECM scaffolds to enhance the physical and biological properties, respectively. These studies certainly provided important knowledge to further optimize the design of ECM scaffolds, but the study of ECM material itself was less reported.

As ECM scaffolds are made from native tissues, their biological properties are primarily depended on the original sources, which quality can be varied by different species, gender, age, weight, health status etc. Among various factors, the age of donor tissue could be one of the most significant variables in ECM’s biological properties [16]. The distinct differences have been found in the structural, componential, mechanical and biological properties of ECM harvested from animal with different ages [17]. It has been also reported that ECM scaffolds derived from neonatal mouse tissues have better effects on the differentiation of MSCs in skin [18] and foetal cardiac ECM enhance the adhesion and expansion of neonatal cardiomyocytes in cardiac regeneration [19]. Hence, in order to further optimize the ECM scaffolds in cartilage regeneration, it would be important to first understand the intrinsic differences of cartilage ECM derived from animal with different ages, and how these differences would impact the properties of ECM scaffolds and the chondrogenic effects of BMSCs towards a successful regeneration of articular cartilage. In this study, we prepared three different articular cartilage tissues from newborn, juvenile and adult rabbits. The mechanical strength, sulphated glycosaminoglycan (sGAG) and collagen contents were characterized before and after the decellularization. Subsequently, three collagen composite hydrogels with decellularization cartilage tissues from newborn, juvenile or adult rabbits were fabricated and mixed with BMSCs. The differences in three hydrogels’ material-related properties, the chondrogenic differentiation of BMSCs *in vitro*, and the cartilage formation *in vivo* are investigated and compared.

## Materials and methods

### Preparation and characterization of native cartilage tissue

#### Preparation of native cartilage tissue

Articular cartilage tissues were cut from the knee joints of 3-, 100- and 200-day old New Zealand Rabbits (Breeding Farm for Sichuan Provincial Experimental Animal Special Committee) immediately following euthanasia. Cartilage slices were harvested with a razor blade and washed with PBS.

#### Water content

To measure the wet weight (WW) of cartilage tissue, excessive moisture on tissue surface was first removed with filter paper, before placing on an electronic balance (ME204) to record the WW. The same cartilage tissue was then dehydrated at 70°C for 72 h, and the dry weight was measured accordingly. The water content of native cartilage tissue from newborn, juvenile or adult rabbits was calculated as (WW − dry weight)/ WW *100%.

#### Mechanical assessment

The mechanical assessment of each sample was determined using a nano-indenter (Piuma, Optics11, The Netherlands). The effective young’s modulus was calculated by data fitting the following parameters: probe stiffness 4.3 (N m^−1^); tip radius 51.0 μm.

#### Quantitation of DNA

The quantitation of DNA was achieved using the Quant-iT^TM^ Picogreen^TM^ dsDNA assay kit. In brief, cartilage particles were digested in the papain solution (0.1 mg/ml) at 65°C for 24 h before tests. A 100 μl of the papain digestive juice was diluted with TE (1×) and then mixed with Picogreen working liquid. After incubation in dark for 3–5 min, the mixture was measured by fluorometry according to the manufacturer’s protocol.

#### Quantitation of sGAG and total collagen

The sGAG content was determined with the Blyscan GAG assay kit (Biocolor, Newtownabbey, UK). Briefly, 10 μl of the supernatant was mixed with Blyscan dye reagent. After centrifugation, the sGAG–dye complex was dissolved in the dissociation reagent followed by the measurement of absorbance at 656 nm using a Thermo reader (Multiskan FC). The total collagen content was measured using the Woessner hydroxyproline assay [20]. The content of hydroxyproline was determined by colourimetry, and the conversion of total hydroxyproline to collagen was using a multiplication factor of 8.2 [21].

### Preparation and characterization of decellularized cartilage particles

#### Preparation of decellularized cartilage particles

The native cartilage tissue was cut into small pieces with a razor blade before further grinding into small particles using a cryogenic grinder (Scientz-48L). The decellularization procedure was adapted from our previous method [22]. In brief, cartilage particles first were washed three times with hypertonic solution (50 mM Tris-HCl buffer and 1 M NaCl, PH = 8) and hypotonic solution (0.5 M Tris-HCl buffer, PH = 8). Subsequently, the particles were frozen in liquid nitrogen and thawed in a 37°C oven for three times. The particles were then added with the 0.5% Triton X-100 solution for 40 min with constant agitation to destroy cell membranes and washed with water for three times. To further eliminate the DNA, 200 U ml^−1^ DNAse I working solution was added and incubated at 37°C for 12 h. Finally, the particles were immersed in 50 mM Ethylenediaminetetra-acetic Acid (EDTA) solution for 20 min and washed with water for three times. The decellularized cartilage particles (DCPs) were frozen in liquid nitrogen and stored in a freezer at −20°C before use.

#### Removal of DNA

The cartilage particles of native and decellularized samples stained by 4′,6-diamidino-2-phenylindole (DAPI) were compared with confirm the removal of cells. To further validate the effectiveness of DNA removal, the DNA content of each sample was quantitated using the Quant-iT^TM^ Picogreen^TM^ dsDNA assay as described in Section ‘Quantitation of DNA’.

#### Size distribution of DCPs

After decellularization, the DCPs from newborn, juvenile or adult rabbits were observed and photographed by scanning electron microscopy (SEM) under low power microscopy. The particle size of each sample was calculated, and the distributions of DCP size were analyzed by ImageJ software.

#### Characterization of DCPs

To further characterize the influence of decellularization procedure on the different DCPs, sGAG, and total collagen were measured using the Blyscan GAG assay and Woessner hydroxyproline assay respectively, as described in Section ‘Quantitation of SGAG and total collagen’.

### Preparation and characterization of collagen-DCP composite hydrogels

#### Preparation of collagen-DCP composite hydrogels

Collagen I hydrogel solution that extracted and purified from calfskin under a sterilized condition in our laboratory [23] was neutralized by 1 M NaOH solution before mixing with DCPs that were sterilized by ^60^Co γ irradiation (at 25 kGy). The final concentration of collagen was 10 mg ml^−1^ and DCPs in the mixture solution were 20 mg mL^−1^ respectively, and the solution casted in a mould at 37°C to form a collagen-DCP composite hydrogel (Φ 8 mm × h 2.5 mm).

#### Microstructural analysis

Collagen-DCP composite hydrogels were dehydrated with gradient ethanol. The microstructures of different collagen-DCP composite hydrogels from newborn, juvenile or adult rabbits were observed under a field emission SEM (Hitachi S-4800, Japan). Collagen hydrogels with the same dimension and collagen concentration were used as control.

#### Mechanical test

The storage and loss modulus of the collagen hydrogel and three different collagen-DCP composite hydrogels (Φ 8 mm × h 2.5 mm) were determined using a dynamic mechanical analyzer (DMA, TA-Q800, USA) at room temperature and its mechanical properties were measured in multi-frequency mode (1–5 Hz).

#### Degradability

The degradation of collagen hydrogel and three different collagen-DCP composite hydrogels (Φ 8 mm × h 2.5 mm) were determined in the solution of collagenase Type I (Sigma) with 30 μg ml^−1^ at 37°C at different time points. The WW of each sample was recorded at initial time points (WW0), and the rate of degradation at different time points was calculated as (WW0 − WWT)/WW0 × 100%, where WWT was the WW of the sample at different time points.

### Chondrogenic differentiation of BMSCs in the collagen-DCP composite hydrogel *in vitro*

#### Isolation and culture of BMSCs

In brief, under sterile condition, long bones were harvested from sacrificed neonatal New Zealand white rabbit. Bone marrow was flushed out using a syringe with a-MEM (Gibco) containing 20% foetal bovine serum (FBS) and 1% penicillin-streptomycin. The cell suspension was filtered through the cell strainer (BD) to remove tissue fragments, followed by centrifugation. The isolated cells were resuspended and cultured in 10-cm cell culture dishes and cultured in a-MEM containing 20% FBS and 1% penicillin-streptomycin. After 24 h, non-adherent cells were washed away, and the attached cells were cultured until reaching 90% confluence. The BMSCs were passaged and cultured in a-MEM containing 10% FBS and 1% penicillin-streptomycin until the third passage (P3) before used in further experiments.

#### Encapsulation of BMSCs in collagen and collagen-DCP composite hydrogels

Collagen I hydrogel solution was neutralized by 1 M NaOH solution before mixing with DCPs from newborn, juvenile or adult rabbits. The mixture of the collagen-DCP solution was then mixed with BMSCs (P3) to reach a final collagen concentration of 10 mg ml^−1^ and DCP concentration of 20 mg ml^−1^, with the cell density of 3 × 10^6^ ml^−1^. The mixture solution was then casted in a mould at 37°C to form a collagen-DCP composite hydrogel (Φ 8 mm × h 2.5 mm). Collagen hydrogels with the same BMSCs density, collagen concentration, and hydrogel dimension were also fabricated for comparison. The hydrogels were subsequently cultured in an ultra-low adhesion surface plate. The culture medium was prepared with 1% penicillin-streptomycin, 90 μg/ml VC, 0.35 mM L-proline, 1% ITS, 1% non-essential amino acids and 1% FBS; with medium changing interval of 2 days.

#### Gross appearance and cell viability

The gross appearance of each sample was observed at Days 1, 3, 7, 14 and 21. The diameter of samples at different time points was recorded and compared. Fluorescein Diacetate (FDA)/ Propidium Iodide (PI) double staining was utilized for cell viability measurement at Day 3, 7 and 14. In brief, samples were gently rinsed in PBS for 5 min and incubated in dark for 5 min with the FDA-PI (1 μg ml^−1^) dye solution. Each sample was observed and photographed under a confocal laser scanning microscope (CLSM, Zeiss-LSM880).

#### Quantitation of DNA and sGAG

To determine the cell proliferation and sGAG secretion, samples were collected and lyophilized for 48 h in a freeze dryer. The dry weight was measured before digested in a papain solution. The cell proliferation (DNA content at different time points) and the sGAG quantity was analyzed using the Quant-iT^TM^ picogreen^TM^ dsDNA assay and the Blyscan GAG assay, which were previously described in Sections ‘Quantitation of DNA’ and ‘Quantitation of SGAG and total collagen’.

#### Histological and immunohistochemical staining

The BMSCs/hydrogel samples were collected at Days 1, 7, 14 and 21 and fixed with 4% paraformaldehyde. The fixed samples were dehydrated by a gradient of 15% and 30% sucrose. The sample was embedded in the optimal cutting temperature embedding agent and then sectioned (6 μm) in a frozen section machine (Leica, RM2016). The cell and tissue morphology in each sample were observed by Haematoxylin–Eosin (HE) staining, secretion of GAGs was detected by Toluidine blue (TB) staining, and calcium deposition was highlighted with the Alizarin red staining (ARS). Furthermore, immunohistochemical (IHC) of collagen Type II (COL2A1) was performed with the mouse monoclonal Collagen II antibody (1:200, Novus Biologicals, NB600-844) primary antibody and the goat anti-mouse IgG (H + L) HRP (1:200, Affinity, S0002) was the secondary antibody for COLII. In brief, the sections were blocked with goat serum for 2 h to block the non-specific antigen, before incubated with the primary antibodies at 4°C for 10 h. After washed three times with TBS, the sections were immersed with 0.3% H_2_O_2_ for 10 min to deplete endogenous peroxidase, followed by incubation with the secondary antibody for 20 min. The sections were coloured with diamino-benzidine staining solution and re-dyed with haematoxylin. The sections were dehydrated by gradient ethanol and treated with xylene solution. Finally, the sections were sealed with neutral gum and observed under a light microscope (LEICA DM1000).

### Cartilage formation of BMSCs in the collagen-DCP composite hydrogel *in vivo*

#### Subcutaneous implantation in nude mice

BMSCs in collagen and collagen-DCP composite hydrogel (Φ 8 mm × h 2.5 mm) were prepared as described in Section ‘Encapsulation of BMSCs in collagen and collagen-DCP composite hydrogels’, and pre-cultured *in vitro* for 3 days. Subcutaneous implantation in nude mice experiments strictly abided by the rules and regulations of the Sichuan University Ethics Committee. In brief, 24 male BALB/c nude mice (6 weeks old) were purchased from GemPharmatech Co. Ltd. After intraperitoneal injection with 10 mg ml^−1^ pentobarbital sodium, nude mice in anaesthesia condition were placed on an ultra-clean table covered with sterile sheets. The back skin of a nude mouse was disinfected with povidone–iodine, and ophthalmological scissors were used to cut a ∼1.5 cm incision on the back of a nude mouse. Four pre-cultured BMSCs/hydrogel constructs were implanted into two subcutaneous pockets before the skin incision was closed with 4-0 needle suture and disinfected with povidone–iodine. Finally, the nude mice were sent back to their original cages, before euthanasia at day 14 and 28. The implants were collected at each time points for further investigation.

#### Biochemical analysis

The gross appearance of each implant was observed before (Day 0), and 14/28 days after implantation. The diameter of samples at different time points were recorded and compared. The DNA and sGAG quantity in each sample were analyzed before (Day 0) and 14/28 days after the implantation using the Quant-iT^TM^ picogreen^TM^ dsDNA and Blyscan GAG assays that described in Sections ‘Quantitation of DNA’ and ‘Quantitation of sGAG and total collagen’. Histological staining of HE, TB and ARS, and the IHC staining of COL2 were performed on each implant at Days 0, 14 and 28. The detailed methodologies were described in Section ‘Histological and immunohistochemical staining’.

#### Gene expression analysis

The mRNA expression levels of four chondrogenic genes (Aggrecan, COL2A1, SOX9 and collagen Type X [COL10A1]) in each implanted construct were analyzed at Days 14 and 28 using Quantitative Real-time PCR (qRT-PCR) technique. In brief, samples were washed with PBS and grinded in an enzyme-free tube with lysate. RNA was extracted using the RNeasy^®^ Mini Kit (Qiagen) and the RNA concentration was measured using a spectrophotometer (ND1000, Nanodrop Technologies). The isolated RNA was reverse transcribed into cDNA using the iScript^TM^ cDNA Synthesis Kit (BIO-RAD). Finally, the cDNA in RNAse-free water was mixed with Ssofast EvaGreen Supermix (BIO-RAD) and the upstream and downstream primers, and the qRT-PCR was performed using the CFX96^TM^ qRT-PCR detection system (Bio-Rad, USA). The genes expression was determined by the software of BioRad CFX Manager, which calculated the relative gene expression based on the ΔΔCt method using the housekeeping gene of GAPDH. The forward and reverse primers’ sequences were referred from previous literature [24] and summarized in Table 1.

**Table 1:** The forward and reverse primer sequences for qRT-PCR

Gene	Forward primer sequences (5′ -3′)	Reverse primer sequences (5′ -3′)
GAPDH	TCGGAGTGAACGGATTTGGC	TTCCCGTTCTCAGCCTTGAC
COL2A1	TGATAAGGATGTGTGGAAGCCG	CAGGCAGTCCTTGGTGTCTTC
Aggrecan	GGCCACTGTTACCGTCACTT	GTCCTGAGCGTTGTTGTTGAC
SOX9	TCTGGAGACTGCTGAACGAG	CTGCCCATTCTTCACCGACTT
COL10A1	TCCCAGA ACCCAGAATCCATC	GGTTGTGGGCCT TTTATGCC

### Statistical analysis

The quantitative data involved in this experiment were presented as mean ± SD. The experimental data were compared by the t-test and the analysis of variance test method using SPSS software. *P < 0.05 was considered to have a significant difference, **P < 0.01 and ***P < 0.001 were considered to be a higher level of significant difference.

## Results and discussion

### Comparison of native cartilage tissue from rabbits with different ages

#### Water content and mechanical strength

**Figure 1:**
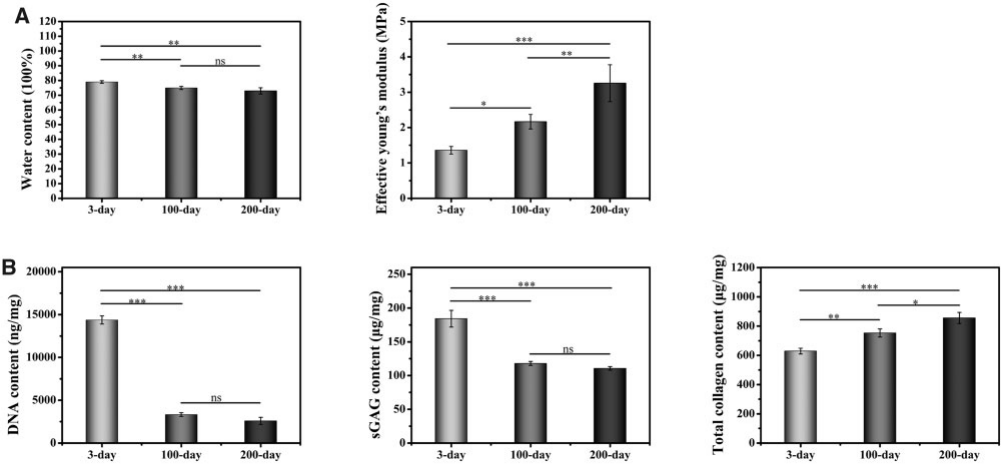
Characterization of native cartilage tissue from newborn, juvenile or adult rabbits. (**A**) Water content (left) and mechanical strength (middle). (**B**) DNA, sGAG and total collagen content (*n* = 3). ns indicates *P* > 0.05, * indicates *P* < 0.05, ** indicates *P* < 0.01 and *** indicates *P* < 0.001.

The differences in the native cartilage tissue from newborn, juvenile or adult rabbits were first compared. The water content (Fig. 1A) in 3-day old rabbit’s cartilage tissue was significantly higher than the other two samples. There was no significant difference in water content between 100- and 200-day. The mechanical property determined by the effective young’s modulus demonstrated the mechanical strength of cartilage tissue was increased with age, and the difference between each group were statistically significant.

#### Quantitation of DNA, sGAG and total collagen

Cell density in the cartilage tissue from newborn, juvenile or adult rabbits was assessed by the quantitation of DNA content (Fig. 1B), which showed a decreasing trend with age. The DNA content was significantly higher in the 3-day sample at 14 371 ± 480 ng/mg, which was over four times more than the other two samples. The DNA content in the 100-day sample was 3325 ± 220 ng/mg, which is higher than the 2588 ± 410 ng/mg in the 200-day samples, but this difference was not statically significant. The sGAG content in three different groups followed the same trend of DNA, with the value of 184.17 ± 12.30, 117.87 ± 2.97, and 110.57 ± 2.61 μg/mg in the 3-, 100- and 200-day samples respectively. The sGAG value in the 3-day sample was statistically higher than the other two samples, but the difference between 100- and 200-day samples was not statically significant. In contrast, the total collagen content showed a reversing trend to the DNA and sGAG values in three groups, where the 200-day sample had the highest value of 855.51 ± 38.14 μg/mg, followed by 753.53 ± 27.69 μg/mg in the 100-day sample and 629.10 ± 19.68 μg/mg in the 3-day sample. Notably, the differences in total collagen content among all groups were statistically significant.

### Characterization of DCPs derived from rabbits with different ages

#### Effectiveness of decellularization

**Figure 2:**
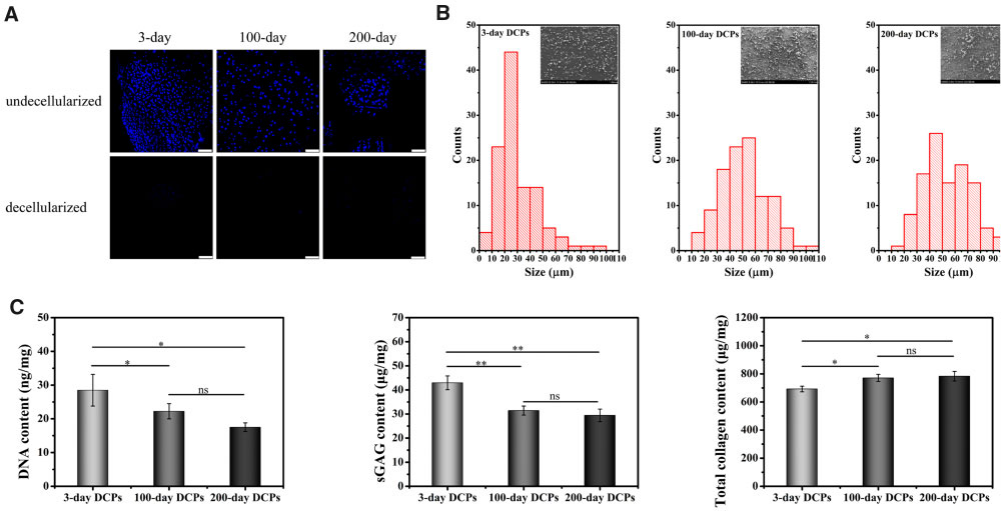
Characterization of DCPs from newborn, juvenile or adult rabbits. (**A**) DAPI staining before and after decellularization. Scale bars represent 100 μm. (**B**) Size distributions of the DCPs. (**C**) The quantification of DNA, sGAG and total collagen in the DCPs (*n* = 3). ns indicates *P* > 0.05,* indicates *P* < 0.05, ** indicates *P* < 0.01 and *** indicates *P* < 0.001.

To evaluate the effectiveness of our decellularization process, DAPI staining of nuclei was applied to cartilage tissues before and after decellularization and compared in Fig. 2A. Nuclei were stained in blue before the decellularization (top panel) and not visually visible after the decellularization (bottom panel). To further validate the effectiveness of decellularization, DNA concentration in each decellularized sample was quantitated. The results (Fig. 2C, left) showed that DNA content were 28.48 ± 4.70, 22.23 ± 2.26 and 17.50 ± 1.30 ng/mg in the 3-, 100- and 200-day samples; representing the decellularization rate of 99.80%, 99.33% and 99.32% respectively. Although the term decellularization has not been defined by quantitative metrics, <50 ng dsDNA per mg ECM dry weight were reported to satisfy the objective of decellularization [25].

#### Grain size distribution

The DCPs from newborn, juvenile or adult rabbits were further characterized. First, the grain size of different DCPs was analyzed, and the size distribution chart with photographs was shown in Fig. 2B. The DCPs derived from 3-day samples had the smallest size, which mainly ranged from 20 to 30 μm. In contrast, the grain sizes of DCPs derived from 100- to 200-day samples were mainly in the range of 30–60 μm.

#### Quantitation of sGAG and total collagen

The sGAG concentration (Fig. 2C, middle) demonstrated a statistically higher sGAG concentration of 42.94 ± 2.85 μg/mg in the 3-day DCPs, while the 31.43 ± 1.88 μg/mg in the 100-day DCPs and 29.43 ± 2.60 μg/mg in the 200-day DCPs were not statistically different. In contrast, total collagen content (Fig. 2C, right) was the lowest in the 3-day DCPs, with the value of 692.46 ± 19.83 μg/mg, which was significantly lower than the concentration of 770.75 ± 25.35 and 783.20 ± 34.10 μg/mg in the 100- and 200-day DCPs, respectively.

### Preparation of collagen-DCP composite hydrogels

#### Gross appearance and microstructure

**Figure 3:**
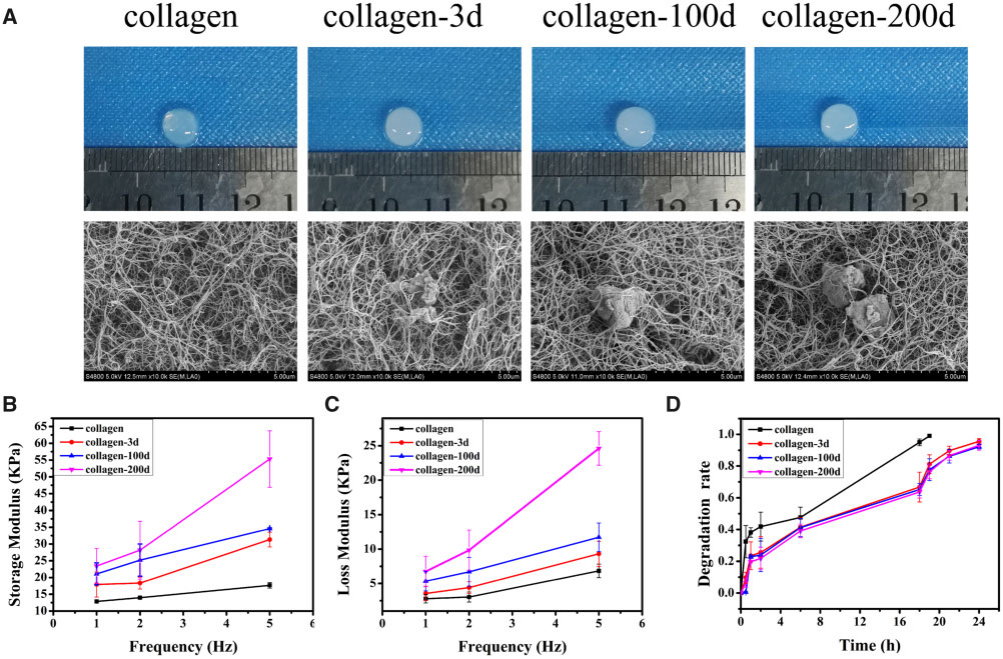
(**A**) Gross appearance and SEM of collagen and collagen-DCP hydrogels. (**B**) Storage modulus and (**C**) loss modulus of collagen and collagen-DCP hydrogels. (**D**) The degradation rate of collagen and collagen-DCP hydrogels.

The collagen and three collagen-DCP composite hydrogels were fabricated and shown in the top panel of Fig. 3A. Collagen hydrogels presented more translucent appearance, while the collagen-DCP composite hydrogels made from 3-day DCPs (collagen-3d), 100-day DCPs (collagen-100d) and 200-day DCPs (collagen-200d) all presented more ivory colour, indicating the incorporation of DCPs. The microstructures (Fig. 3a, bottom panel) by SEM showed that the thickness and network pattern of collagen fibres were looked similar in all four hydrogels, showing the preservation of collagen fibre thickness and network structure in the composite hydrogels. Furthermore, it was clear that individual DPCs were entrapped in the collagen network.

#### Mechanical properties and degradation

The mechanical properties were characterized by the storage modulus (Fig. 3B) and loss modulus (Fig. 3C), which respectively represented the elasticity and viscosity of a hydrogel. In general, both storage and loss modulus were the lowest in collagen hydrogel and increased with the incorporation of DCPs. Moreover, with the DCPs derived from older rabbits, both storage and loss modulus of the composite hydrogel increased further. As a result, the overall storage and loss modulus of four hydrogels followed the order of collagen-200d > collagen-100d > collagen-3d > collagen. The degradation rates of four hydrogels in collagenase solution were shown in Fig. 3D. In general, collagen hydrogel had a higher degradation rate, with the complete degradation within 19 h. In contrast, all three collagen-DCP composite hydrogels had a similar degradation curve, with the degradation percentages of 95.56%, 92.20% and 93.07% in the collagen-3d, collagen-100d and collagen-200d hydrogels at 24 h, respectively.

### The chondrogenic differentiation of BMSCs in collagen-DCP composite hydrogels *in vitro*

#### Gross appearance and cell viability

**Figure 4:**
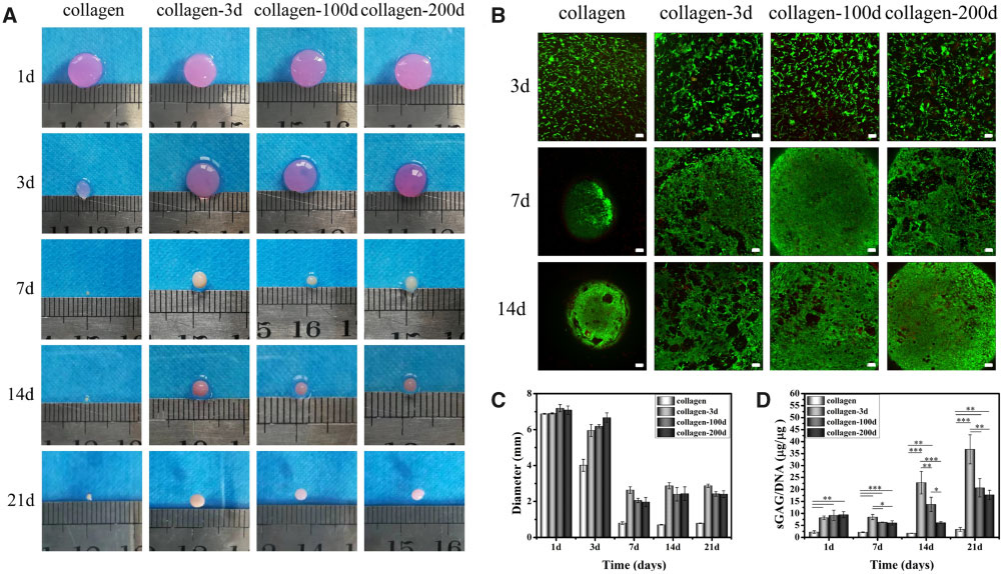
(**A**) Gross appearance of four BMSCs/hydrogel constructs. (**B**) FDA/PI image of four hydrogels from Days 3 to 14. Scale bars represent 100 μm. (**C**) The diameter change and (**D**) quantitative analysis of sGAG/DNA in each sample at different time points. Data are presented as the mean ± SD (*n* = 3). ns indicates *P* > 0.05, * indicates *P* < 0.05, ** indicates *P* < 0.01 and *** indicates *P* < 0.001.

BMSCs were encapsulated in the four different hydrogels, and the gross appearances of four BMSCs/hydrogel constructs were photographed at different time points throughout the *in vitro* culture (Fig. 4A). Despite the same initial size at Day 1, the four BMSCs/hydrogel constructs underwent different levels of contraction with extended culture, and the diameters at different time points were shown in Fig. 4C. Among the four samples, the collagen hydrogel with BMSCs had the highest contraction at Day 3, and its diameter almost halved from the original size. In contrast, the diameter of the other three samples was not changed much (<10%). At Day 7, the contraction was further progressed in the collagen hydrogel sample, with only 0.80 ± 0.07 mm in diameter. Clear contractions were also observed in the three collagen-DCP composite hydrogel samples, with the diameters reduced to 2.64 ± 0.19, 2.06 ± 0.12 and 1.97 ± 0.26 mm in collagen-3d, collagen-100d and collagen-200d samples, respectively. The contraction wasn’t further progressed at Day 14, and the sizes of three collagen-DCP composite hydrogel samples were slightly increased from Days 7 to 14, indicating the possible matrix secretion in these three samples. After that, the size of all samples almost remained the same on Day 14. At the end of *in vitro* culture, the average diameters of four hydrogel samples were in the order of collagen-3d > collagen-100d ≈ collagen-200d > collagen. Despite the clear contraction of four hydrogel samples, the live/dead staining images (Fig. 4B) showed that BMSCs were uniformly distributed and maintained high cell viability (>95%) in four hydrogels at alltime points, and the number of cells was visually increased with time. Together, it demonstrated that all four hydrogels have good cytocompatibility to BMSCs.

#### Chondrogenic differentiation of BMSCs

**Figure 5:**
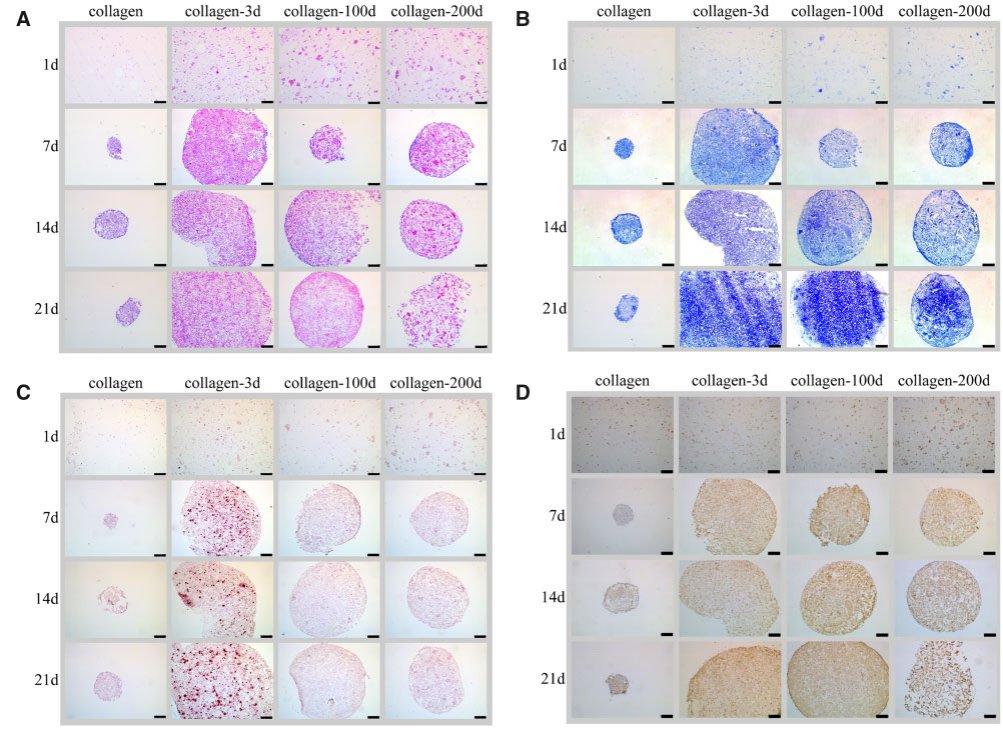
Representative images of four hydrogels after culture with BMSCs. (**A**) HE staining, (**B**) TB staining, (**C**) ARS staining and (**D**) IHC staining of COL2. Scale bars represent 250 μm.

The chondrogenic differentiation of BMSCs in four different hydrogels was first assessed by comparing the production of sGAG, which is the key matrix protein in articular cartilage. The quantitation of sGAG/DNA (Fig. 4D) showed that the incorporation of DCPs significantly increased the sGAG/DNA secretion in all three collagen-DCP composite hydrogel samples, and the quantitation of sGAG/DNA was in the order of collagen-3d > collagen-100d > collagen-200d > collagen at the end of 21-day culture *in vitro*. The result was further validated by the histological and IHC staining of the samples. HE (Fig. 5A) staining showed the homogenous distribution of cells in all four samples on Day 1. The individual DCPs were also visible in three collagen-DCP composite hydrogel samples, with the size of DCPs in collagen-3d was smaller than the ones in collagen-100d and collagen-200d samples. Similarly, TB staining (Fig. 5B) also showed the more intensive colour of GAG secretion in three collagen-DCP composite hydrogel samples, with the order of collagen-3d > collagen-100d > collagen-200d and the lowest TB staining was shown in the collagen hydrogel sample. Furthermore, the IHC staining of COL2 (Fig. 5D), the most abundant collagen in native cartilage, was shown a higher staining intensity in the collagen-3d and collagen-100d samples, followed by the collagen-200d samples, and the lowest staining was found in the collagen hydrogel sample. Together, both TB and COL2 staining showed a similar trend, which indicated that DCPs derived from younger rabbits resulted in better chondrogenic differentiation of BMSCs. However, the ARS staining (Fig. 5C) of calcium deposition presented a clear staining in the collagen-3d samples, indicating the possible osteogenic differentiation of BMSCs in this sample.

### Cartilage formation of BMSCs in collagen-DCP composite hydrogels *in vivo*

#### Gross appearance

**Figure 6:**
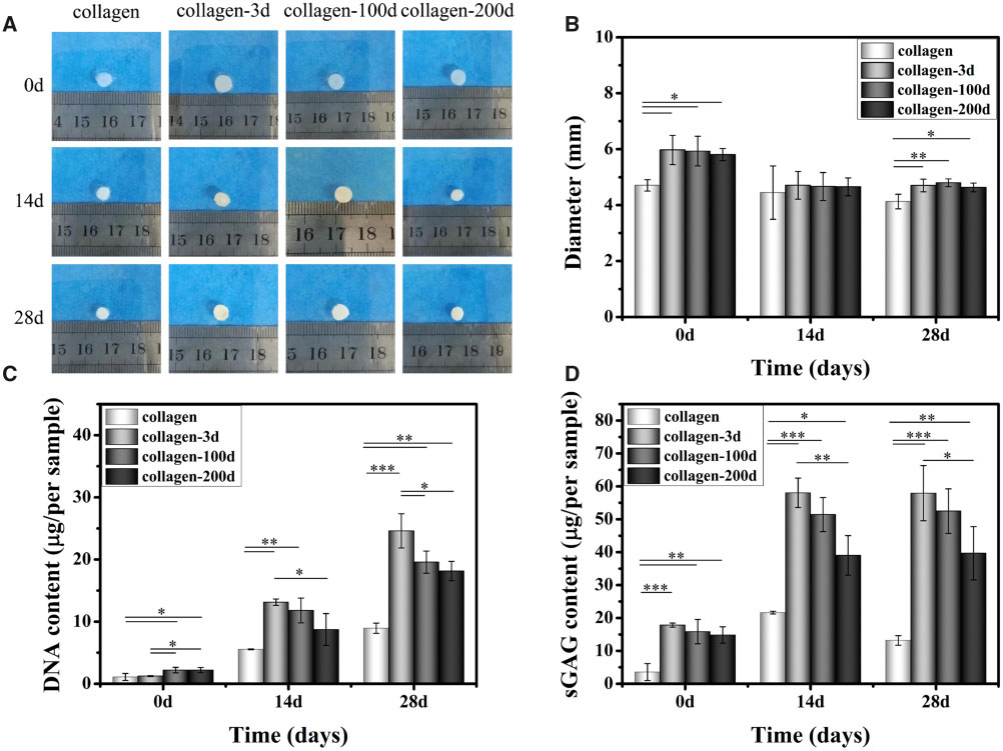
(**A**) The gross appearance of four BMSCs/hydrogel constructs before and after 14/28 days’ implantation *in vivo*. (**B**) The diameter change, quantitative analysis of (**C**) DNA and (**D**) sGAG synthesis in each sample at different time points. Data are expressed as the mean ± SD (*n* = 3). ns indicates *P* > 0.05, * indicates *P* < 0.05, ** indicates *P* < 0.01 and *** indicates *P* < 0.001.

The gross appearance of four BMSCs/hydrogel constructs before and after 14/28 days’ implantation was shown in Fig. 6A. Due to the 3-day pre-culture *in vitro*, the collagen sample underwent a greater contraction than the other three samples (Fig. 6B) and was ∼1 mm smaller in diameter than others before the implantation. However, the collagen sample size almost remained the same after 14 days’ implantation, but a slight decrease in size was observed in all the other three samples. Together, the diameter of all four samples had no statistical difference on Day 14. With extended culture, there was no significant difference in the diameters of all four samples cultured to 28 days compared with 14 days.

#### Quantitation of DNA and sGAG

Due to the 3-day pre-culture *in vitro*, different initial DNA and sGAG content were found in various samples before the implantation (Fig. 6C and D). After 14- and 28-day’s implantation *in vivo*, the DNA and sGAG content in four samples followed a similar trend. In general, the collagen sample had the lowest DNA and sGAG production, which was significantly lower than the other three samples at alltime points. Among three collagen-DCP composite hydrogel samples, the collagen-3d sample achieved the most DNA and sGAG concentration, followed by the collagen-100d sample, and the collagen-200d sample was the lowest at both Days 14 and 28.

#### Chondrogenic genes expression

**Figure 7:**
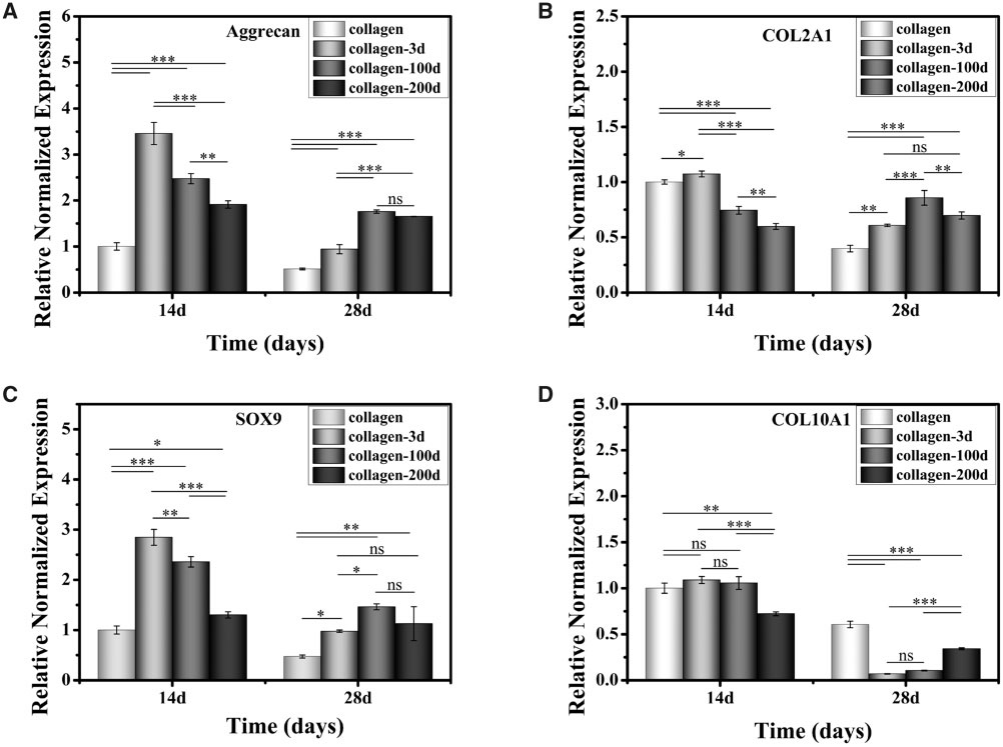
Cartilage-related genes expression of (**A**) Aggrecan, (**B**) COL2A1, (**C**) SOX9 and (**D**) COL10A1. Data are presented as the mean ± SD (*n* = 3). ns indicates *P* > 0.05, * indicates *P* < 0.05, ** indicates *P* < 0.01 and *** indicates *P* < 0.001.

Figure 7 shows the expression of chondrogenic genes in four groups of samples on Days 14 and 28 *in vivo*. Aggrecan and COL2A1 genes are specific matrix related markers of hyaline cartilage. SOX9 is a marker gene of chondrocyte phenotype, and COL10A1 is a gene associated with chondrocyte hypertrophy. Aggrecan and SOX9 showed a similar trend, with the expression level of collagen-3d > collagen-100d > collagen-200d > collagen on Day 14, and collagen-100d > collagen-200d > collagen-3d > collagen on Day 28. The COL2A1 expression was high in collagen-3d and collagen samples on Day 14, but decreased on Day 28. In contrast, the COL2A1 expression was lower in the collagen-100d and collagen-200d samples on Day 14, but slightly increased on Day 28. The hypertrophic marker gene of COL10A1 exerted a similar level among the collagen, collage-3d and collagen-100d samples on Day 14, but they all decreased on Day 28. The results suggested that the collagen hydrogel with DCPs could not only promote the expression of genes associated with chondrogenic differentiation but also inhibited the hypertrophy.

#### Histological and IHC staining

**Figure 8:**
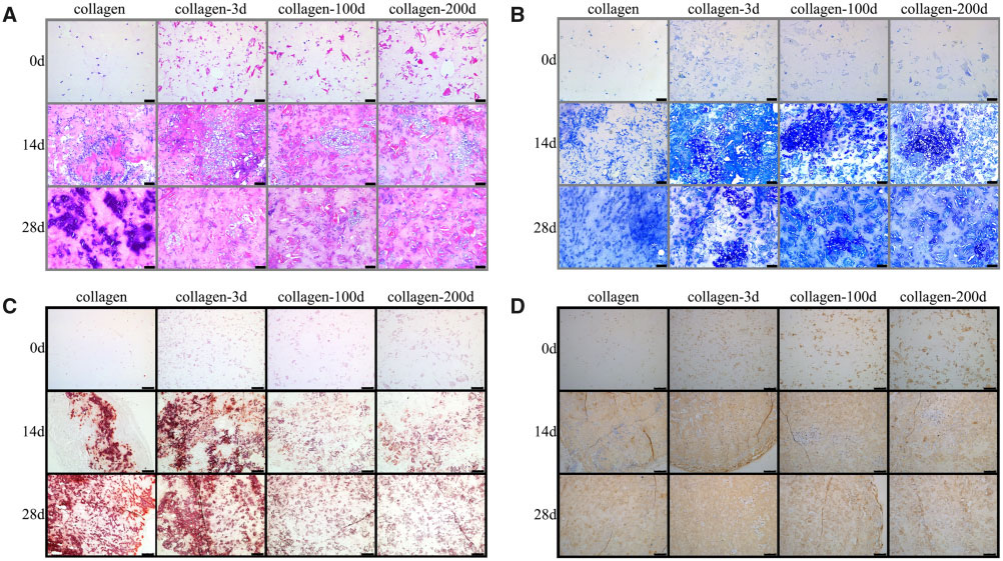
Representative images of four BMSCs/hydrogel before and after 14/28 days’ implantation *in vivo*. (**A**) HE staining and (**B**) TB staining. Scale bars represent 100 μm. (**C**) ARS staining and (**D**) IHC staining of COL2. Scale bars represent 250 μm.

The cartilaginous tissue formation *in vivo* was further investigated using histological and IHC staining. HE staining (Fig. 8A) showed more and larger chondrocyte clusters in collagen-DCP composite hydrogels, while most of the BMSCs encapsulated in collagen hydrogels presented smaller cell clusters which presumably inflammatory cells. TB staining (Fig. 8B) showed more intensive staining in the collagen-3d sample, followed by the collagen-100d and collagen-200d samples, and the collagen sample had the lowest staining among all four groups. The IHC staining of COL2 (Fig. 8D) was observed in all four samples, but the collagen-3d and collagen-100d samples had visually more intense staining than the collagen-200d and collagen samples, indicating the better COL2A1 production in the earlier two samples. Notably, intensive ARS staining (Fig. 8C) was observed in both collagen and collagen-3d samples. It showed that the BMSCs in these two groups might underwent osteogenic differentiation and therefore the newly synthesized matrix was calcified.

## Discussion

When using native cartilage tissue as raw materials to fabricate ECM scaffold, it is important to understand the intrinsic properties of cartilage tissues, since this may provide crucial information to further optimizing the selection of ECM materials. This study was designed to characterize the decellularized cartilage ECM from newborn, juvenile or adult rabbits, and their influences on the chondrogenic differentiation of BMSCs and cartilaginous ECM synthesis over a 21-day culture *in vitro* and 28-day implantation *in vivo*. To our knowledge, this is the first study that directly compared the ECM scaffolds made from cartilage tissues with different ages, and how the intrinsic properties of decellularized tissue with different ages would impact the properties of ECM scaffolds, which further influence their chondrogenic performance towards a successful cartilage regeneration. Our study presents several notable findings on the chondrogenic effects of ECM scaffolds made from three cartilage tissues with different ages. We showed that the mechanical strength, sGAG and total collagen contents were statistically different among three ECM scaffolds made from cartilage tissues with different ages, and consequently affected the cell proliferation and differentiation among three samples. Detailed biochemical and gene analysis showed that the ECM scaffolds made from younger cartilage tissues led to better chondrogenesis of BMSCs, but unfortunately the newborn cartilage tissue would also result in the unwanted tissue calcification.

The componential and mechanical differences of articular cartilage tissues from newborn (3-day), juvenile (100-day) and adult (200-day) rabbits were first compared. It was found that the DNA and sGAG content in the 3-day cartilage tissue was statistically higher than the 100- and 200-day samples [26, 27]. sGAG are negatively charged polysaccharides that bind and retain water and water-soluble molecules [28]. Consequently, sGAG content of the sample correlated to its water content, which was the highest in the 3-day sample, and showed a decreasing trend with age [29]. In contrast, the total collagen content in articular cartilage was found to increase with rabbits’ age [30]. Collagen is the most abundant structural macromolecule in cartilage tissue, and provides the main resistance to stress [31]. Hence, the total collagen contents of cartilage tissue were in-line with the mechanical strength, which showed an increasing trend with age. The decellularized procedure has effectively removed more than 99% of dsDNA in all cartilage tissues with different ages, and the dsDNA concentration are all below the critical decellularization level of <50 ng/mg dry weight [10]. As sGAG in ECM were attached to the collagen fibre network through weak intermolecular interactions such as non-covalent bonds [32], which could be easily interrupted during the decellularization procedure. Hence, only 25–30% of the sGAG remained in DCPs after decellularization. In contrast, total collagen contents were almost unchanged in all three DCPs after decellularization, indicating that the decellularization process would not lead to the degradation or removal of collagen network structure, and retained the basic structure of cartilage tissue. These results were also consistent with the previous studies [9, 33]. Together, DCPs derived from three cartilage tissues with different ages remained the decreasing trend of sGAG and increasing trend of total collagen content with age. After the same grinding process, the cartilage tissue with higher mechanical strength resulted in larger grain size of cartilage tissue particles. Consequently, 3-day DCPs had the smallest, and 200-day DCPs had the largest grain size. Although the size difference didn’t affect the effectiveness of decellularization, it might impact the cell attachment and behaviours on the DCPs [34].

Three ECM scaffolds, named collagen-3d, collagen-100d, and collagen-200d hydrogels, were made by mixing of collagen hydrogel and different DCPs with different ages. The SEM images demonstrated that the collagen network was preserved well in all samples, with individual DCPs entrapping in the collagen network. Hence, the mechanical strength of individual collagen-DCP hydrogel was influenced by the added DCPs, and the storage and loss modulus of three hydrogels were followed the order of collagen-200d > collagen-100d > collagen-3d. When culture with BMSCs, the additional DCPs also delayed the degradation and reduced the shrinkage of hydrogels *in vitro*, and the age-depended performance of chondrogenic differentiation of BMSCs and sGAG synthesis in three hydrogels was shown both *in vitro* and *in vivo*. The collagen-3d hydrogel achieved the most chondrogenesis, followed by collagen-100d and the collagen-200d was the least. This finding confirmed that the ECM scaffolds from newborn, juvenile or adult rabbits would lead to different chondrogenic inducibility to BMSCs. It could be due to the combination of many factors. First, due to the higher sGAG concentration in the younger sample, more sGAG was able to preserve in the ECM scaffolds derived from younger rabbits. The natural cartilage ECM is rich in GAGs, which mainly consists of hyaluronan acid, chondroitin sulphate, dermatan sulphate, keratan sulphate and heparan sulphate. So far, many studies have confirmed their functions in the regulation of chondrogenesis [35] and the addition of GAGs in biomaterials could promote the chondrogenic differentiation of stem cells. Hence, the higher sGAG content in younger DCPs would be beneficial in the chondrogenesis of BMSCs. Besides, due to the lower mechanical strength of younger cartilage tissue, DCPs derived from younger rabbits have smaller grain size, with homogenous dispersion in the collagen-DCP composite hydrogel. When mixing with BMSCs, smaller ECM particles have been reported to enhance cell-material interaction, and improve the induction ability of cartilage ECM-derived scaffolds [36]. Moreover, the mechanical property of hydrogels also influenced the chondrogenesis of BMSCs. Due to the increasing mechanical strength of DCPs with rabbits’ age, the mechanical strength of collagen-DCP hydrogels also increased. Bian *et al*. [37] found that the low crosslinking density hydrogels with lower mechanical strength were more conducive to the formation of cartilage matrix compared with the high crosslinking constructs. Nevertheless, tissue calcification is a common problem when utilizing BMSCs for cartilage regeneration *in vitro* and *in vivo* [38, 39]. This problem was found in collagen and collagen-3d samples in this study, it probably due to different reasons. The tissue mineralization in collagen hydrogel *in vivo* was due to its fast degradation property that led to the loss of structural supports for cells, which consequently couldn’t maintain the chondrogenic phenotype before the neo-tissue structure was formed [40]. In contrast, the collagen-3d had much lower degradation than collagen and was almost identical to collagen-100d and collagen-200d. In this case, the tissue calcification induced by collagen-3d could be due to its intrinsic property. As cartilage serves as a template for bone growth from the embryonic stage to skeletal development [41], the cartilage ECM during this stage also influence the development of the cultured cells and induced the osteogenesis of BMSCs. Hence, the collagen-3d scaffold derived from newborn ECM inherited this function and resulted in the calcification of regenerated matrix. In contrast, the collagen-100d and collagen-200d scaffolds are derived from juvenile and adult ECM, which no longer involves the endochondral bone formation process and are not facilitate the osteogenic differentiation of BMSCs. In the context of this finding, it will be interesting to further compare the detailed compositional and biological differences among cartilage tissues with different ages in developmental biology.

## Conclusion

In this study, articular cartilage tissues from newborn, juvenile and adult rabbits were harvested and decellularized. The mechanical strength, sGAG and collagen contents were statistically different among three aged samples before and after decellularization. Consequently, the composite hydrogels made of different DCPs with different ages demonstrated age-dependent chondrogenic inducibility. In general, the newborn DCPs promoted the most chondrogenesis of BMSCs but led to severe matrix calcification. In contrast, the juvenile and adult DCPs had less cartilaginous matrix production but no visible osteogenesis was observed. Together, it highlighted the importance of age differences when using native cartilage tissues to fabricate ECM scaffolds in cartilage tissue engineering. Although the newborn sample has the best chondrogenic effects, it also demonstrated clear osteogenic effects, which would be a crucial shortcoming to limit its application in cartilage regeneration. In contrast, the juvenile sample achieved the best overall results in promoting chondrogenesis and inhibiting osteogenesis of BMSCs. Consequently, juvenile ECM could be a better source than both newborn and adult ECMs. The findings provide important information to our current knowledge in the design of future ECM-based biomaterials towards a successful repair of articular cartilage.
